# CLPREM: A real-time traffic prediction method for 5G mobile network

**DOI:** 10.1371/journal.pone.0288296

**Published:** 2024-04-01

**Authors:** Xiaorui Wu, Chunling Wu

**Affiliations:** 1 National Key Laboratory of Wireless Communications, University of Electronic Science and Technology of China, Chengdu, Sichuan, China; 2 School of Artificial Intelligence and Big Data, Chongqing College of Electronic Engineering, Chongqing, China; University of Carthage National School of Engineers of Carthage (ENICarthage) / Higher School of Communications of Tunis (Sup’Com), TUNISIA

## Abstract

Network traffic prediction is an important network monitoring method, which is widely used in network resource optimization and anomaly detection. However, with the increasing scale of networks and the rapid development of 5-th generation mobile networks (5G), traditional traffic forecasting methods are no longer applicable. To solve this problem, this paper applies Long Short-Term Memory (LSTM) network, data augmentation, clustering algorithm, model compression, and other technologies, and proposes a Cluster-based Lightweight PREdiction Model (CLPREM), a method for real-time traffic prediction of 5G mobile networks. We have designed unique data processing and classification methods to make CLPREM more robust than traditional neural network models. To demonstrate the effectiveness of the method, we designed and conducted experiments in a variety of settings. Experimental results confirm that CLPREM can obtain higher accuracy than traditional prediction schemes with less time cost. To address the occasional anomaly prediction issue in CLPREM, we propose a preprocessing method that minimally impacts time overhead. This approach not only enhances the accuracy of CLPREM but also effectively resolves the real-time traffic prediction challenge in 5G mobile networks.

## 1 Introduction

In recent years, with the rapid deployment and development of the Internet of Things, mobile traffic has exploded, according to Ericsson’s latest Mobility Reports, at the end of 2021, global mobile data traffic was 67 exabytes per month, and will increase 4.2 times in the next five years to about 282 exabytes per month [1]. 4-th generation mobile networks (4G) networks are facing the challenge of huge mobile traffic, and will gradually be unable to meet the massive access needs of the Internet of Things [2]. 5G is a new technological revolution to meet the explosive growth of mobile communication demand and has greatly promoted the digitalization, networking and intelligence of the economy and society [3]. Compared to the 4G network, the 5G network offers significant advantages, such as ultra-high speed, ultra-reliability, ultra-short delay, and massive connection capabilities. Additionally, by enhancing the quality of service (QoS), it effectively addresses the requirements for extensive mobile traffic forwarding, thereby expediting the progression of the Internet of Everything [4]. To solve the huge traffic load caused by massive heterogeneous data on traditional cellular networks, 5G operators deploy a large number of low-power micro-base stations around the macro base station to offload the macro base station and achieve the purpose of load balancing. Therefore, to optimize the deployment and allocation of 5G mobile network resources in large-scale cities, improve the intelligence and reliability of traffic management, and deeply understand the traffic characteristics of 5G mobile networks, it is important to predict traffic with high accuracy and low complexity [5].

A time series is a chronological sequence of statistical indicators, capturing the progression of a phenomenon over time. Time series forecasting involves analyzing the patterns, direction, and trends exhibited by the time series data, and making predictions about future levels or trends based on extrapolation or extension techniques. It aims to infer the future development process and anticipate the potential outcomes in the next period or in the years to come. It can be seen that 5G network traffic is essentially time series data, so its prediction problems can be transformed into time series forecast modeling problems. For example, [6] analyzes the network traffic of a large number of cellular base stations, distinguishes the traffic into predictable and unpredictable parts, and proves that predictable traffic has self-correlation. [7] proposes a Seasonal Autoregressive Integrated Moving Average (SARIMA) model, which accurately captures the seasonal characteristics of network traffic by analyzing the autocorrelation of time series, and then obtains long-term traffic prediction results. In order to investigate the potential impact of the number of surrounding base stations on the traffic of cellular base stations, [8] proposes the use of an *α*-stable model to predict network traffic considering both temporal and spatial aspects. The experimental results demonstrate that the proposed model achieves improved prediction accuracy compared to linear models such as Autoregressive Integrated Moving Average (ARIMA) and SARIMA. However, with the continuous expansion of cellular networks in terms of width and depth, the characteristics of network traffic have diverged significantly from the assumptions of linear prediction models mentioned above. Consequently, subsequent research has shifted towards nonlinear prediction models within the field of statistics [9]. In recent studies [10, 11], various nonlinear prediction models such as the Multifractal Wavelet Model (MWM), the Autoregressive Conditional Heteroskedasticity (ARCH) model, and the Fractal Autoregressive Integrated Average Moving Average (FARIMA) model have been employed to forecast network traffic. These models are capable of capturing the intricate nonlinear characteristics of network traffic, surpassing the accuracy achieved by linear models. However, despite their improved performance, the prediction accuracy still falls short of meeting the demand.

Whether in the past or now, some researchers have been obsessed with improving statistical models in an attempt to achieve more accurate predictions of network traffic, but experiments have proved that most of these algorithms are not powerful enough, mainly for the following two reasons:

- The linear model is too simple. Even if only a single communication protocol is used for data transmission, the relationship between future network traffic and past network traffic is difficult to describe as a linear function. Not to mention the large number of communication protocols used in the actual communication process of 5G mobile networks, the number of packets of different protocols, and the probability distribution of packet lengths are different, which brings great difficulties to the real-time estimation of 5G mobile network traffic. These complex relationships are difficult to describe with linear models, so it is almost impossible to accurately predict traffic by building a linear model alone.
- Nonlinear model parameters are difficult to estimate and the accuracy is still not high. With the same number of model parameters, accuracy also improves relative to the linear model capturing more nonlinear relationships between network traffic. Unfortunately, accurately estimating model parameters for nonlinear models requires significant computational resources [12]. Although researchers can often mathematically approximate model parameters when certain conditions are true, this approximate parameter estimation method does not always hold when estimating complex 5G mobile traffic. This results in most nonlinear models either having high time complexity or not substantially improving accuracy compared to linear models.

In recent years, with the rapid development of big data collection technology and artificial intelligence technology, machine learning has gradually become a popular direction for non-parametric predictive models [13], which has been favored by more researchers. [14] compared the performance of various statistical models, including the Last-Value Predictor (LVP), Autoregressive Model (AR), Moving Average Model (MA), Double Exponential Smoothing (DES), Autoregression Moving Average (ARMA), as well as Artificial Neural Network (ANN) and Wavelet Transform (WT) models, in predicting real-time application traffic, using metrics such as computation cost, storage cost, and accuracy. Finally, it is pointed out that the ANN prediction model is always more effective than other prediction schemes for various application traffic prediction schemes, but it also consumes more computing and storage resources, so the prediction scheme based on the ANN model should be selected when the computing and storage resources are sufficient.

In response to the problem of mobile network traffic prediction, [15] used a Convolutional Neural Network (CNN) to extract deep features from the time series, and then input a Recurrent Neural Network (RNN) to improve the accuracy of traditional RNN network predictions. The characteristics of network traffic, such as nonlinearity, long-range correlation, time-varying, self-similarity, and burstiness, can easily lead to slow convergence and premature convergence of the gravitational search algorithm. To address this issue, [16] improved the gravitational coefficient and velocity selection formula, enhanced the global search capability of the model, and applied the improved gravitational search algorithm optimized Radial Basis Function Neural Network (RBF) to predict Lorenz chaotic time series and network traffic, achieving higher accuracy than traditional RBF network. The Temporal Convolutional Network (TCN) can fully extract and utilize the historical information of time series data to achieve more accurate predictions than traditional neural networks [17]. Therefore, [18] proposed a city network traffic prediction framework based on the TCN model, which can accurately capture the spatiotemporal evolution of traffic and apply it to traffic prediction across the whole city, with prediction accuracy improved by 15.14% and 14.64%.

To address the problem of slow convergence and easily falling into local minimums of traditional neural networks in network traffic prediction, [19] used the Partial Least Squares (PLS) fast pruning algorithm to simplify the network topology, which improved the convergence speed and accuracy of the model. To simultaneously predict traffic for multiple nodes in a mobile network, previous studies [20, 21] have adopted a method of combining the traffic time series from multiple nodes into a high-dimensional matrix. They applied various algorithms such as Multi-Layer Perceptron (MLP), Multi-Layer Perceptron with Weight Decay (MLPWD), and Support Vector Machines (SVM) for prediction. This approach enables the prediction of traffic patterns across multiple nodes in a mobile network. The study found that SVM is more accurate in predicting high-dimensional traffic data, while MLPWD has higher accuracy in predicting low-dimensional traffic data. In addition, using high-dimensional traffic data for prediction can significantly improve the prediction accuracy of MLP, MLPWD, and SVM algorithms.

There are many studies using deep learning to predict network traffic, most of which are based on training models according to the temporal characteristics of collected traffic data and using them to predict future traffic. These methods are generally feasible in some scenarios, but if directly applied to solve the problem of real-time prediction of 5G mobile network traffic, the following problems are likely to exist:

- The model is too simple to make accurate predictions. The amount of useful information input to a deep learning model often determines the upper limit of the model’s accuracy, and adjusting the model and its parameters can make the model approach the limit of accuracy. However, 5G mobile network traffic has complex characteristics such as self-similarity, burstiness, and periodicity. If only a small portion of historical traffic data is used for prediction, the prediction results will heavily rely on the historical data and it will be difficult to obtain accurate results.
- The model has a high computational complexity, making it difficult to ensure real-time prediction. The model extracts too many traffic data features, making the model framework very large and the training process complex, and the model difficult to converge. Even successfully trained models may only learn surface features of the data and experience overfitting. Most importantly, overly complex models consume a lot of computing resources and time, making it difficult to ensure real-time prediction.
- The model has poor generalization ability. The data set used to train the model is too coarse, and there is no specific feature classification for the samples, only concatenating features together as the data set, resulting in low-quality training samples and poor generalization of the model [22].

We propose CLPREM to address the real-time traffic prediction problem in 5G mobile networks discussed above. We evaluate the performance of CLPREM in terms of accuracy using root mean square error (RMSE), assess its timeliness by calculating the prediction time for a single step, and evaluate its generalization ability by comparing the average errors across multiple environments. The main contributions of this paper are as follows:

- We propose CLPREM, a robust, accurate, and lightweight model. CLPREM is based on the LSTM network, combined with data augmentation, clustering algorithms, and a heuristic lightweight method, effectively addressing various challenges in real-time prediction of 5G mobile network traffic.
- To enhance the robustness and generalization of the model, we design a time-series generator based on Time series Generative Adversarial Network (TimeGAN), which can generate an additional time series based on the temporal characteristics of the original time series and add it to the training dataset. We validate the effectiveness of this approach by calculating the temporal similarity between the generated and original time series.
- We design a traffic classifier based on clustering algorithms to differentiate traffic data from different network behaviors, and conduct experiments to verify the robustness of the traffic classifier, further improving the accuracy and generalization ability of the model.
- In addition, we propose a heuristic lightweight algorithm for CLPREM to reduce time consumption while maintaining a comparable level of accuracy, achieving the goal of real-time prediction.
- Finally, we introduce a preprocessing method that almost does not increase computational complexity to alleviate the tailing problem of CLPREM in predicting highly fluctuating time series, further improving the accuracy of the model.

The remaining content of this paper is organized as follows: The System Overview section briefly introduces the components of the model and the training and prediction process. The System Design section provides a detailed explanation of the functions of each module and the related technologies used. In the Experimental Results Analysis section, we present the experiments designed to validate the model’s rationality. To evaluate the performance of the proposed model, we compare CLPREM with other models based on accuracy and time consumption as the main indicators. To address the tailing phenomenon of the distribution function, we introduce a unique preprocessing method and compare the impact of preprocessing on model accuracy. Finally, in the Conclusion section, we provide a comprehensive and objective evaluation of the proposed model based on the comparison results in section four.

## 2 System overview

CLPREM mainly consists of five modules:

1. The data collection module is responsible for collecting and processing network traffic data in 5G mobile communication and integrating the processed data into a vector as the raw dataset.
2. The data augmentation module uses the TimeGAN network to generate additional datasets based on the raw dataset obtained by the data collection module and adds them to the original dataset to obtain an expanded dataset.
3. The traffic classification module clusters the dataset into several categories based on different network behaviors, used for the training and prediction of the deep learning module.
4. The deep learning module uses the output of the traffic classifier to predict the real-time trends of 5G mobile traffic.
5. The lightweight module compresses the deep learning network to improve its efficiency.

**Fig 1:**
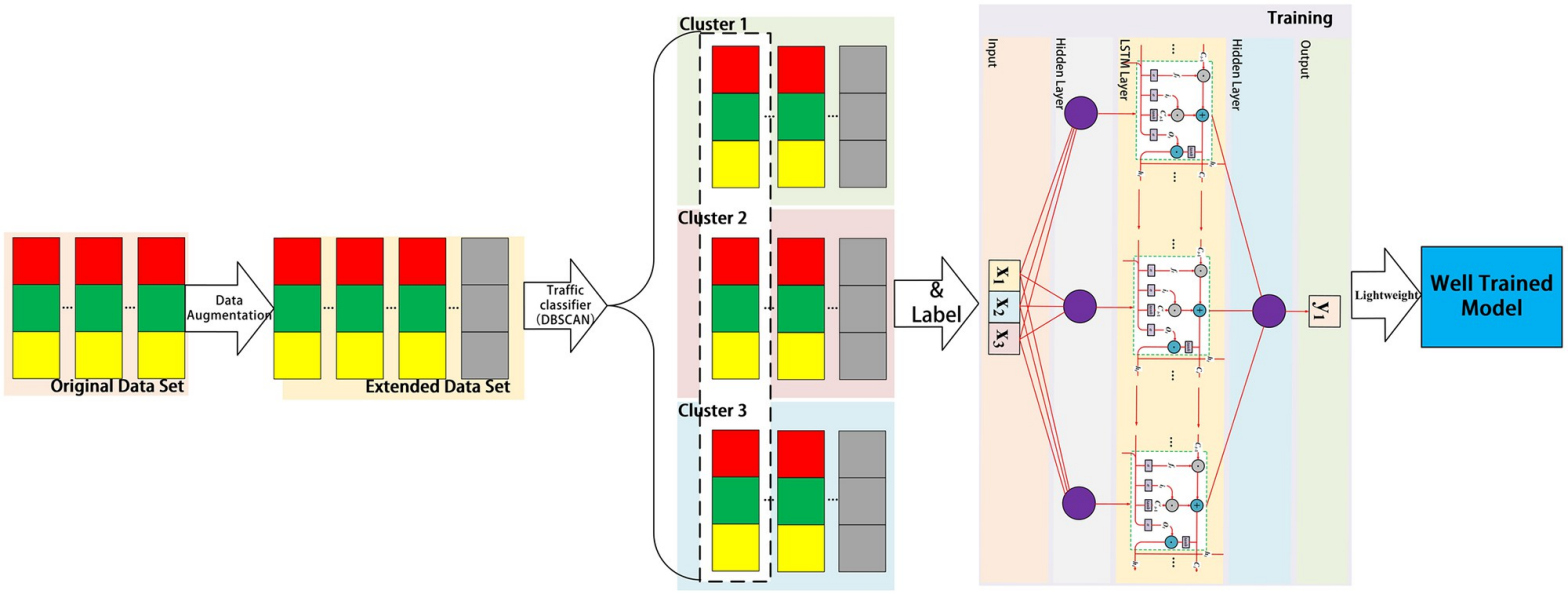
The process of CLPREM training involves expanding the raw dataset, clustering, filling, and labeling the data before inputting it into the prediction module. After model compression, a well-trained CLPREM can be obtained.

Fig 1 shows the training process of CLPREM. Firstly, the data collection module gathers network traffic data and forms samples in the form of vectors, which constitute the original dataset. The original dataset is then fed into the data augmentation module (a time series data generator), and the output is added to the original dataset to obtain an expanded dataset. Next, during the model training phase, the network traffic classification module uses the Density-Based Spatial Clustering of Applications with Noise (DBSCAN) to classify network traffic. By clustering network traffic data generated by different network behaviors into different clusters and adjusting the clustering algorithm’s parameters, the clustering results in only three clusters. The missing data is filled in, and three vector time series with the same length are generated for each cluster. Finally, the three vector sequences are labeled and used as input to the deep learning prediction module for training, with forward propagation predicting results and backward propagation updating weights. After the training is completed, the model is compressed using a lightweight algorithm, and the model is fine-tuned by training it again. The resulting model is the trained CLPREM.

**Fig 2:**
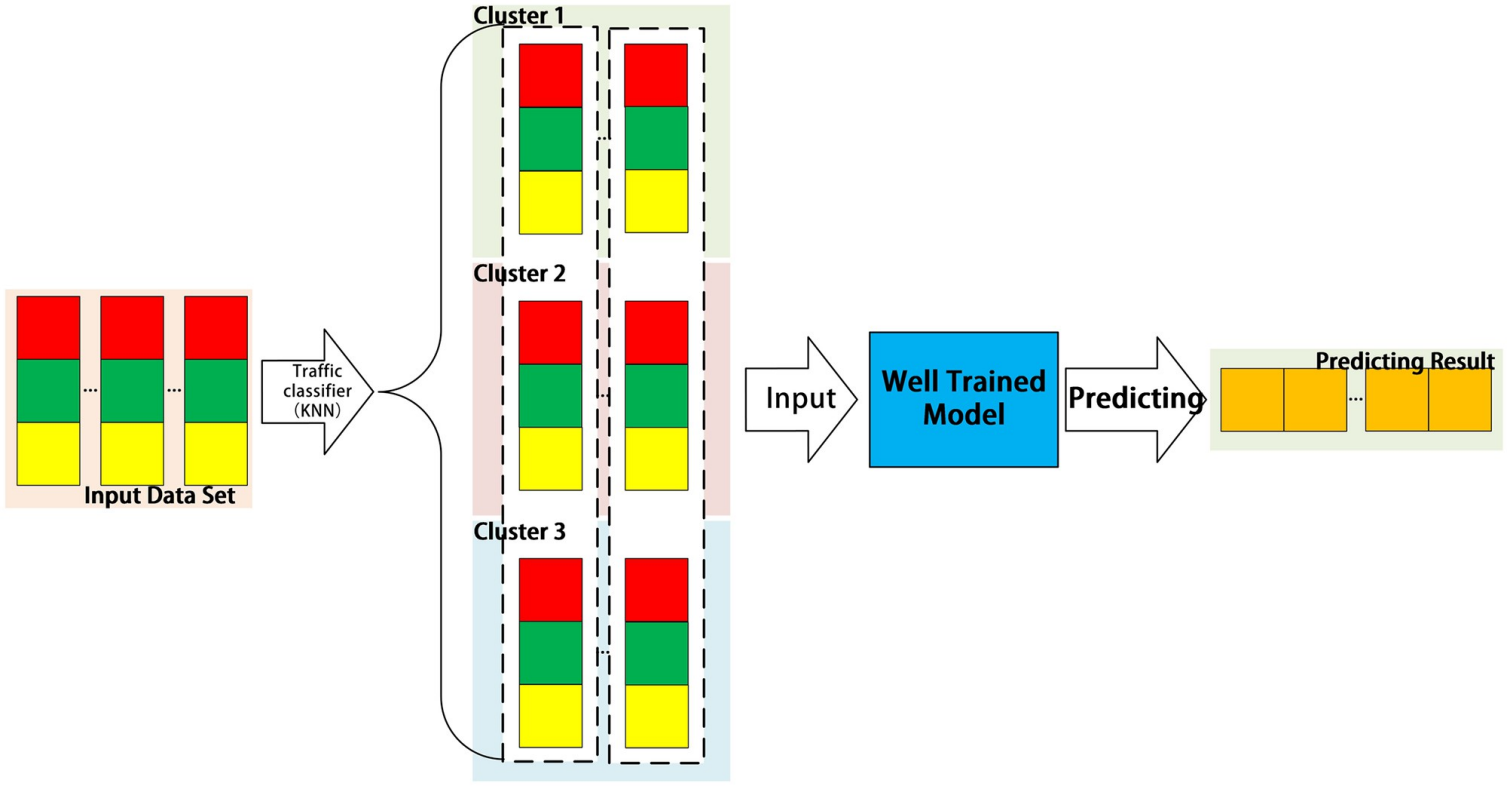
The process of CLPREM prediction. The input dataset represents a batch of input data that needs to be predicted. After undergoing clustering and padding, it is inputted into the trained prediction module. After the prediction is completed, a batch of predicted data is outputted.

Fig 2 shows the prediction method of CLPREM. Real-time data collected by the data collection module is organized into an input dataset using the same method as during model training. The input dataset is then passed through a network traffic classification module where, in consideration of timeliness, the k-Nearest Neighbors (KNN) clustering algorithm is used. After filling the data of each cluster, three time series of equal length are obtained and input into the well-trained prediction module, ultimately producing a batch of predicted data. This section briefly explains the training and prediction methods of CLPREM. In the System Design section of Chapter 3, the specific operations of the modules involved in the CLPREM training and prediction processes will be detailed.

## 3 System design

### 3.1 Data collection module

The 5G mobile traffic generated by mobile devices is usually composed of many different network traffic, and the transmission protocol types and parameter settings used by this traffic are not the same. Therefore, analyzing 5G mobile traffic based solely on throughput cannot distinguish application types, protocol types, and the characteristics of individual flows, resulting in relatively single data collection and difficulty for the model to learn the essential features of the traffic [23].

Generally, network application services mostly choose Transmission Control Protocol (TCP) or User Datagram Protocol (UDP) as the transport layer protocol. Among them, TCP is a connection-oriented protocol with reliable transmission characteristics, while UDP is a connectionless transport protocol. In the early days, TCP traffic accounted for a large part of mobile network traffic, but in recent years, the proportion of UDP traffic has increased rapidly with the development of new network services. This phenomenon arises from the high data redundancy observed in services like audio communication, video communication, and online gaming. These services are relatively unaffected by the loss of individual data packets but place significant emphasis on real-time performance. They strive to minimize the utilization of management resources while accommodating a larger number of concurrent online users. As a result, these emerging network services increasingly opt for UDP as their underlying transport protocol [24]. The traffic generated by the two main transmission protocols, TCP traffic and, UDP traffic, often exhibit different statistical characteristics. Based on this, this article will additionally statistics TCP and UDP traffic when collecting traffic raw data [25].

To achieve higher accuracy, it is not only necessary to collect data based on requirements, but also to design a suitable data model to carry the collected data to adapt to the input of the deep learning network. This article defines the sample $\vec{V}$ as a vector in a three-dimensional space.

$$\begin{array}{c} \vec{V}=\left(Traffic,TCP_{traffic},UDP_{traffic}\right) \end{array}$$ (1)

Among them, the three coordinates of sample vector $\vec{V}$ represent the value of network traffic during the measurement interval and the TCP and UDP network traffic rates within it.

### 3.2 Data augmentation algorithm

Deep Generative Models (DGMs) have been proven to be able to output datasets that are similar to the input data. In DGMs, Generative Adversarial Networks (GANs) are commonly used to generate synthetic samples and effectively increase the training set [26]. However, the data generated by traditional GANs does not consider the time correlation specific to time series. To address this issue, [27] designed TimeGAN, a data generator based on GANs that considers the time characteristics of time series. TimeGAN combines unsupervised learning methods with supervised learning methods to generate sequence data with time characteristics, often used as a means of data augmentation for time series.

**Fig 3:**
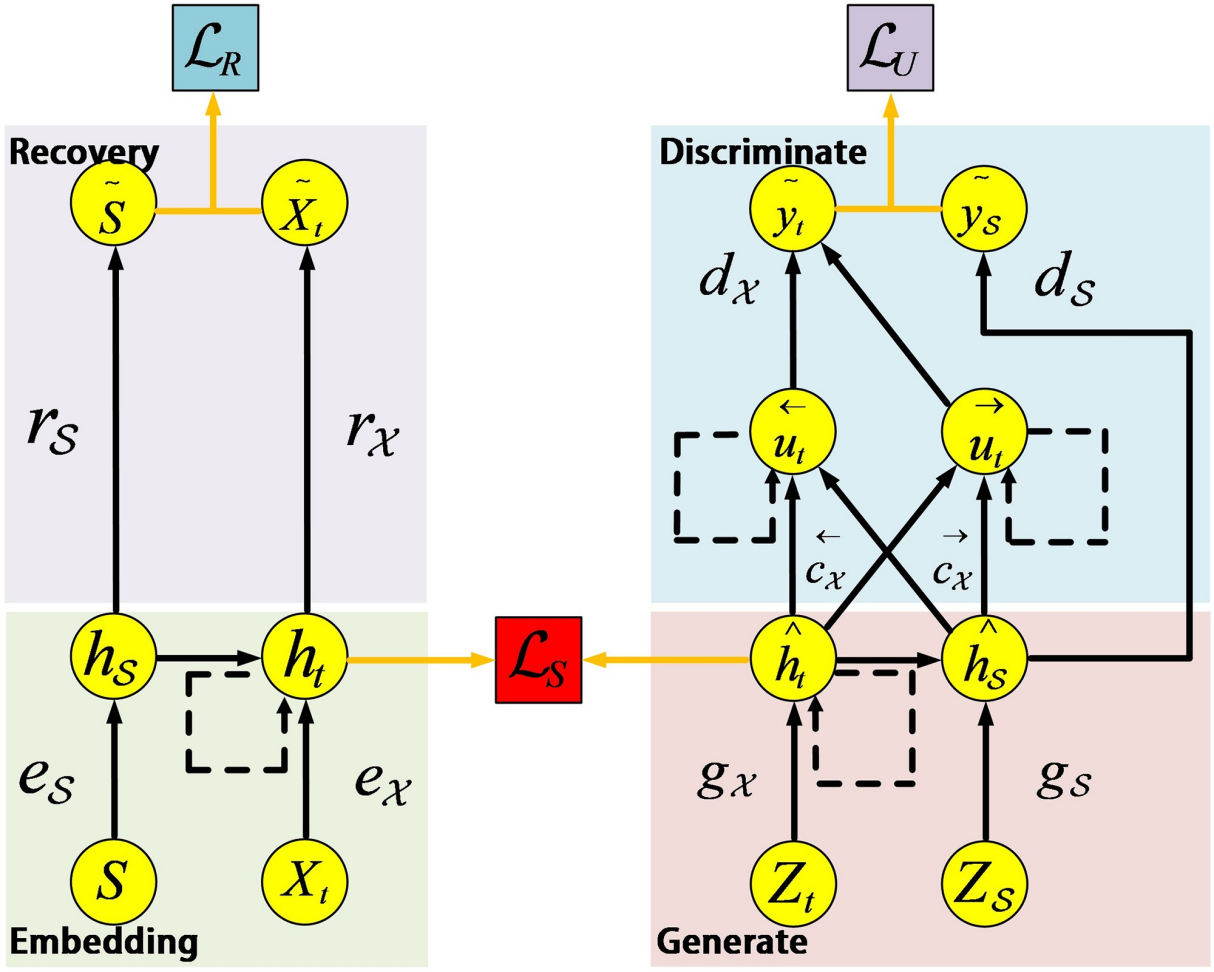
TimeGAN instantiated with RNN, solid lines denote function application, dashed lines denote recurrence, and orange lines indicate loss computation.

As shown in Fig 3, TimeGAN consists of four network components: Embedding, Recovery, Generator, and Discriminator, which essentially let the model learn the probability distribution of the time series from the training dataset. TimeGAN believes that time series should have two characteristics, the first is a static feature, which does not change due to time, and the second is a time series feature, which changes over time. TimeGAN represents features through vectors, and defines **S** and **X** as vector spaces for static features and dynamic features, respectively, and S and X are samples belonging to the **S** and **X** vector space. By definition, a time series training dataset can be expressed as $D={\left(S_{n},X_{n,1:T_{n}}\right)}_{n=1}^{N}$, TimeGAN realizes the function of using dataset D to train a probability distribution $\hat{p}\left(S,X_{1:T}\right)$ to make it as close as possible to the probability distribution *p*(*S*, *X*_1:*T*_) of the original data, and generate a new dataset D’ on this basis. Although there have been many studies using the TimeGAN algorithm to generate new time series for data augmentation, it still needs further analysis to determine if this method is appropriate for extending 5G mobile network traffic data. To address this issue, this paper plans to compare the similarity between the original sequence D and the sequence D’ generated by TimeGAN using the Dynamic time-warping (DTW) algorithm. The DTW algorithm is based on dynamic programming and measures the similarity between two time series by calculating the dynamic time-warping distance [28]. According to the DTW algorithm, for time series *X* = (*x*_1_, *x*_2_, *x*_3_, … *x*_*m*_) and *Y* = (*y*_1_, *y*_2_, *y*_3_, … *y*_*m*_), the dynamic time-warping distance of X and Y is defined as follows:

$$\begin{array}{c} D(<>,<>)=0 \end{array}$$ (2)

$$\begin{array}{c} D(X,<>)=D(<>,Y)=\infty \end{array}$$ (3)

$$\begin{array}{c} D(X,Y)=D(x_{1},y_{1})+min\left\{D\left(X,Rest\left(Y\right)\right),D\left(RestX,Y\right),D\left(RestX,Rest\left(Y\right)\right)\right\} \end{array}$$ (4)

$$\begin{array}{c} (x,y)=|x-y|,\;\;\;\;x\in X,y\in Y \end{array}$$ (5)

In the above formula, <> represents an empty sequence, *Rest*(*X*) = (*x*_2_, *x*_3_, … *x*_*m*_), *Rest*(*Y*) = (*y*_2_, *y*_3_, … *y*_*n*_), and *d*(*x*, *y*) represents the distance between x and y. The problem of solving the dynamic time-warping distance can be understood as using the idea of dynamic programming to find a curved path with the minimum warping cost.

### 3.3 Traffic classifier module

After adding the samples generated by data augmentation to the original dataset, an extended dataset can be obtained, which can be directly used for model training. However, this method cannot distinguish between different network behaviors, and cannot capture the changing trends of TCP and UDP traffic, making it difficult to further improve the accuracy of the model. In order to improve the prediction accuracy and generalization ability of the model, this article plans to use the DBSCAN algorithm to divide the extended dataset into several categories to distinguish different network behaviors during model training. After filling in the time series through padding operations, this is used as the input data for the model.

DBSCAN is a representative density clustering algorithm, whose principle can be summarized as selecting a core point and continuously expanding towards the density-reachable area, thus obtaining a maximized area containing core and boundary points. Any two points in the area are density-connected and can be uniquely determined by any core point [29]. The DBSCAN algorithm searches for clusters by examining the epsilon neighborhood of each object in the dataset. If the number of points in object p’s epsilon neighborhood is less than M, then object p is marked as a non-core object. If it is greater than or equal to M, then a cluster is created with p as the core object, and then objects that are density-reachable from the core object are iteratively clustered. The algorithm ends when no new points can be added to any cluster.

When using the trained model for prediction, in order to reduce the time cost of clustering, this paper applies a supervised learning method KNN on top of the DBSCAN algorithm for classification based on network conditions during the model training. KNN algorithm is a non-parametric classification algorithm that is very simple and widely used in processing large amounts of high-dimensional data. The core idea of the KNN algorithm is to find the K nearest labeled data to each unlabeled data based on distance, and determine the label of the unlabeled data based on the number of labeled data of each class [30].

In order to classify 5G traffic to improve prediction accuracy, this section designs a network traffic classifier that uses the DBSCAN algorithm during training and the KNN algorithm during prediction. To demonstrate the rationality of the design, this paper will further explain the robustness of using the KNN algorithm instead of the DBSCAN algorithm during prediction through verification in Chapter 4.

### 3.4 Traffic prediction module

LSTM is a type of recurrent neural network, also known as a recursive neural network. Compared to RNN, LSTM has significant advantages in solving problems containing time series data because it can learn long-term dependencies [31]. Each unit structure of LSTM contains a forget gate, an input gate, and an output gate, which can selectively decide how to pass the state of the previous layer to the current layer and which information to output in the current layer. The forgetting gate controls the memory unit and decides what to keep and what to forget in the memory unit state. The forget gate calculates the forget rate by activating sigmoid function with new input information *x*_*t*_ and the previous output *h*_*t*−1_, and applies it to the previous memory *C*_*t*−1_ to determine the degree of retention of the previous memory. The specific calculation is shown below:

$$\begin{array}{c} f_{t}\;=\;\sigma \;\left(w_{f}\;\left[h_{t-1},\;x_{t}\right]+bf\right) \end{array}$$ (6)

In which, *f*_*t*_ is the forget rate, *σ* is the sigmoid activation function, *w*_*f*_ and *b*_*f*_ are the weights and biases of the forget gate, *h*_*t*−1_ is the output of the previous time step, *x*_*t*_ is the input of the current time step.

The input gate consists of sigmoid layer, tanh layer, and state layer. The sigmoid layer and tanh layer determines the weight of adopting new input and old memory in the output and update the adopted new memory to the old memory filtered by the forget gate. The state layer updates the old information, specifically updates *C*_*t*−1_ to *C*_*t*_ based on the output of the sigmoid layer and tanh layer. The expression of the input gate is as follows:

$$\begin{array}{c} i_{t}\;=\;\sigma \;\left(w_{i}\;\left[h_{t-1},\;x_{t}\right]+bi\right) \end{array}$$ (7)

$$\begin{array}{c} C_{t}^{\prime }\;=\;tanh\;\left(w_{c}\;\left[h_{t-1},\;x_{t}\right]+bc\right) \end{array}$$ (8)

$$\begin{array}{c} C_{t}\;=\;f_{t}C_{t-1}\;+\;i_{t}C_{t}^{\prime } \end{array}$$ (9)

In which, *i*_*t*_ is the forgetting rate, *w*_*i*_ and *b*_*i*_ are the weights and biases of the input gate, tanh is the activation function, *w*_*c*_ and *b*_*c*_ are the weights and biases of the cell state layer.

The output gate still uses the sigmoid function to act on the latest memory *C*_*t*_ and obtains the output *h*_*t*_ of the neuron. Similar to the forget gate, the output gate also has a filtering function. The output *h*_*t*_ consists of two parts. The first part is *o*_*t*_, which is obtained from the previous output *h*_*t*−1_, current input *x*_*t*_, and sigmoid function. The second part is composed of the hidden state *C*_*t*_ and the tanh function. The corresponding formula is shown below:

$$\begin{array}{c} o_{t}\;=\;\sigma \;\left(w_{o}\;\left[h_{t-1},\;x_{t}\right]+bo\right) \end{array}$$ (10)

$$\begin{array}{c} h_{t}\;=\;o_{t}\;tanh\left(C_{t}\right) \end{array}$$ (11)

where *o*_*t*_ is the forgetting rate, *w*_*o*_ and *b*_*o*_ are the weights and biases of the output gate, and *h*_*t*_ is the output of the hidden layer.

**Fig 4:**
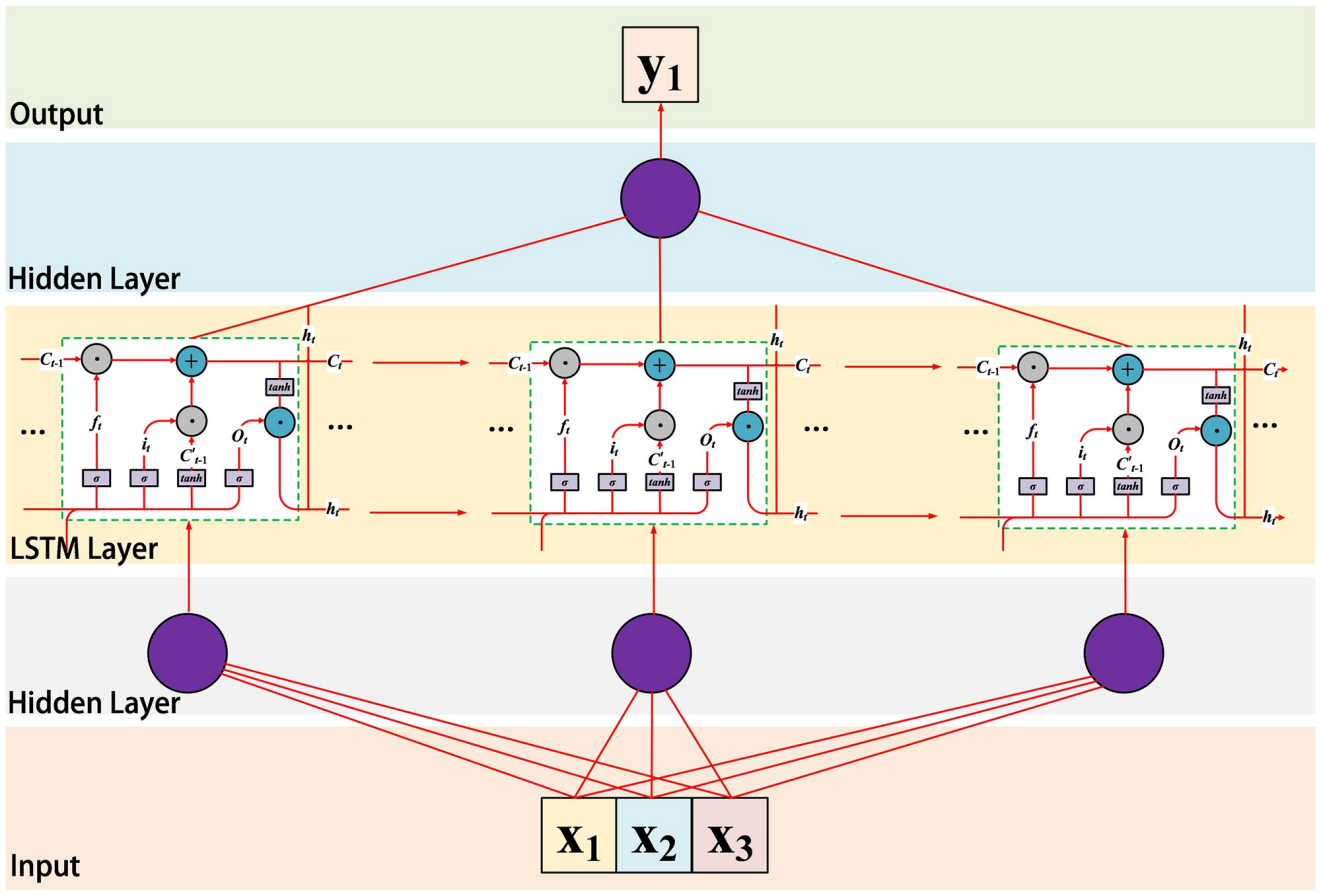
The prediction module of CLPREM consists of three LSTM networks in which the weights are connected to each other. The input data is fed into the LSTM layer through a hidden layer. Then, the output of the LSTM layer is adjusted to obtain the output through another hidden layer.

**Fig 5:**
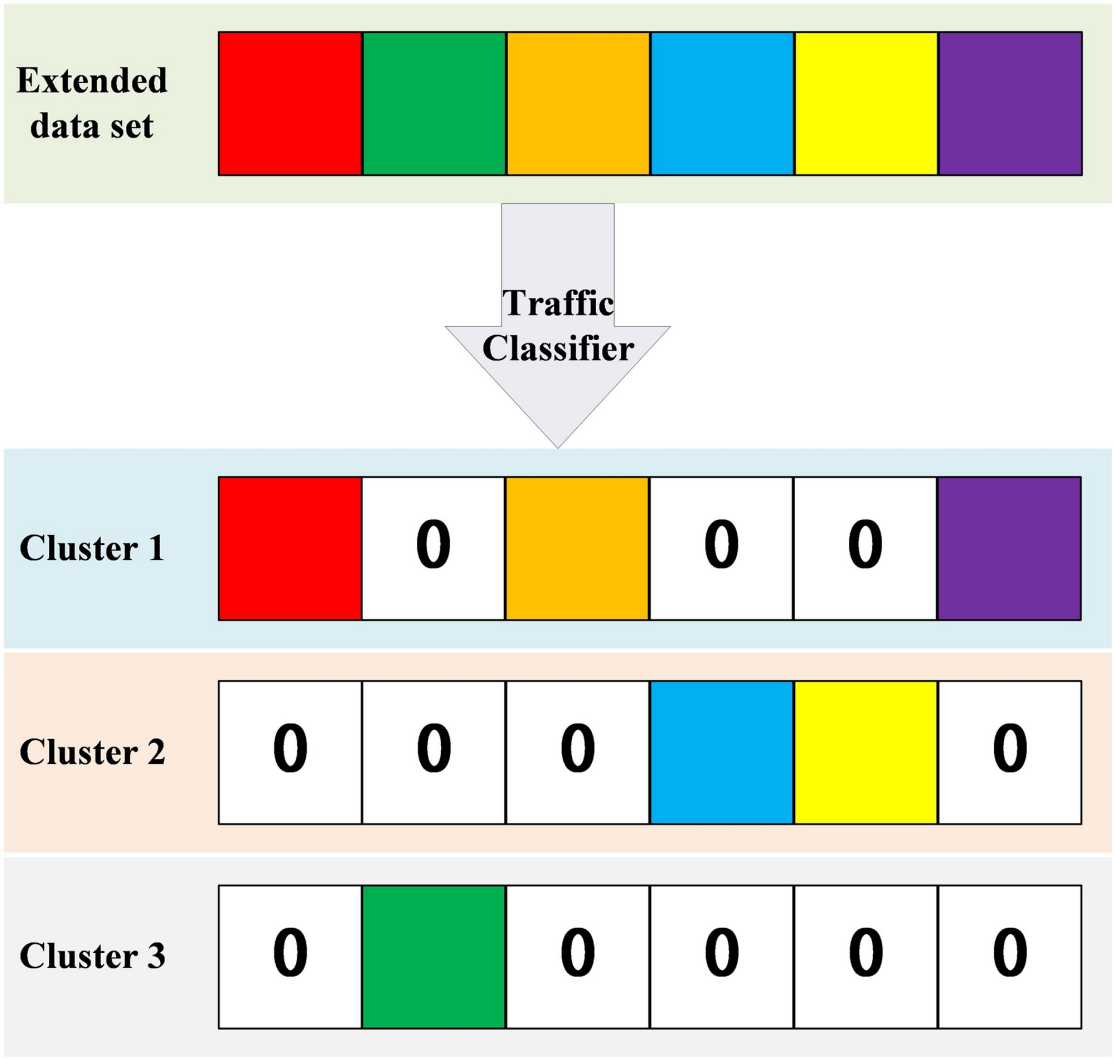
After traffic classification, the traffic classifier completes the filling process for each cluster. The relative positions of each data point remain unchanged, and the missing parts are filled with zeros.

However, the traditional LSTM traffic prediction method mainly predicts the traffic in the next period by measuring the traffic data and its changes over a period of time, where the input time series is *x*_*t*_ = *N*_*i*_, *N*_*i*+1_, …, *N*_*i*+*m*_, (*m* > 0) and the output is *y*_*t*_ = *N*_*i*+*m*_, *N*_*i*+*m*+1_, …, *N*_*i*+*m*+*p*_, (*p* > *m* > 0). Since this method only predicts based on the temporal characteristics of traffic data and does not integrate the application scenarios of 5G mobile networks and other traffic influencing factors, the accuracy of traditional prediction methods is greatly limited. It is prone to congestion, packet loss and other phenomena during traffic surges, while bandwidth waste can occur during traffic declines. To solve this problem, this paper improves the traditional LSTM network and obtains the traffic prediction module shown in Fig 4. The input layer represents several time series of the same length obtained after traffic classification and data padding, which serve as the input data for the prediction module. However, the classification of data by the traffic classifier will inevitably result in the absence of some values in the output time series, and the length of the time series may also be unequal. To avoid this situation and prevent the traffic classifier from destroying the temporal sequence of the sequence, we have designed a data padding method, the process of which is shown in Fig 5. From Fig 5, it can be seen that the time series is divided into multiple non-overlapping subsequences after passing through the traffic classifier. If they are not padded, the time intervals and lengths of each subsequence will be different. To provide equal-length sequences to adapt to the input of the prediction module, each subsequence needs to be padded. The specific method is to fill the empty data with zeros instead of directly concatenating the subsequent data.

After obtaining the input data of the model, a hidden layer completes the mixing of the input data, and the mixed data is input into the prediction module. The prediction module consists of multiple traditional LSTM networks, and the number of networks is the same as the number of input sequences. Finally, the prediction results of each LSTM network are adjusted by another hidden layer to obtain the output, realizing the prediction of traffic.

### 3.5 Lightweight algorithm

Due to the high complexity of deep learning models, using a trained model for prediction often requires significant computational and time resources. In order to achieve real-time prediction, model compression is necessary [32]. Weight pruning determines unimportant weights in the network and removes them to achieve network sparsity, resulting in compression of the network model. This saves time and computational resources and also helps to avoid overfitting, thus improving the model’s generalization ability [33]. In this paper, a heuristic pruning algorithm based on CLPREM is proposed, as shown in Algorithm 1. The algorithm can be divided into three stages. The first stage is pretraining of the initial network, which involves learning the initial weight connections of the network through regular training. The importance of the weights is measured by their absolute value, and those with small absolute values are considered unimportant and removed, while the trained model is saved. The second stage involves pruning the weight connections, removing those with low importance, by calculating the average absolute value of the weights in a layer and removing those below the product of the proportionality coefficient and the mean. This converts the original dense connection network into a sparse one, and the pruned sparse network model is saved. The third stage is fine-tuning the pruned network, by training it again to recover its accuracy. The final weights of the remaining sparse connections are obtained during this stage. The initial training weights are kept during fine-tuning to maintain the connections, rather than initializing them from scratch or randomly. This is because during backpropagation, the network is more likely to find a local minimum close to the initial training weights through gradient descent, which leads to higher accuracy when the pre-trained parameters are used as initial parameters [34].

**Algorithm 1** Proportional pruning algorithm

**Input**: Initial network *W*, proportional threshold *θ*, training dataset *x*, training times *epoch*.

**Output**: Network *W* after proportional pruning.

1: **for** *i* = 1 to *epoch* **do**    ▹ pre-trained Network

2:  *W*_*i*_ = *W*_*i*−1_ − *ρ*∇*f*(*W*_*i*−1_; *x*)

3: **end for**

4: **for** *layer* in *W* **do**       ▹ Weight Pruning

5:  $\bar{w}=\sum_{i=1}^{n}|w_{i}|/n$

6:  **for** *i* = 1 to *n* **do**

7:   **if** $|w_{i}|<\theta \bar{w}$ **then**

8:    *w*_*i*_ = 0

9:   **end if**

10:  **end for**

11: **end for**

12: **for** *i* = 1 to *epoch* **do**     ▹ Re-train Network

13:  *W*_*i*_ = *W*_*i*−1_ − *ρ*∇*f*(*W*_*i*−1_; *x*)

14: **end for**

## 4 Experimental results analysis

### 4.1 Experimental environment and data collection

**Fig 6:**
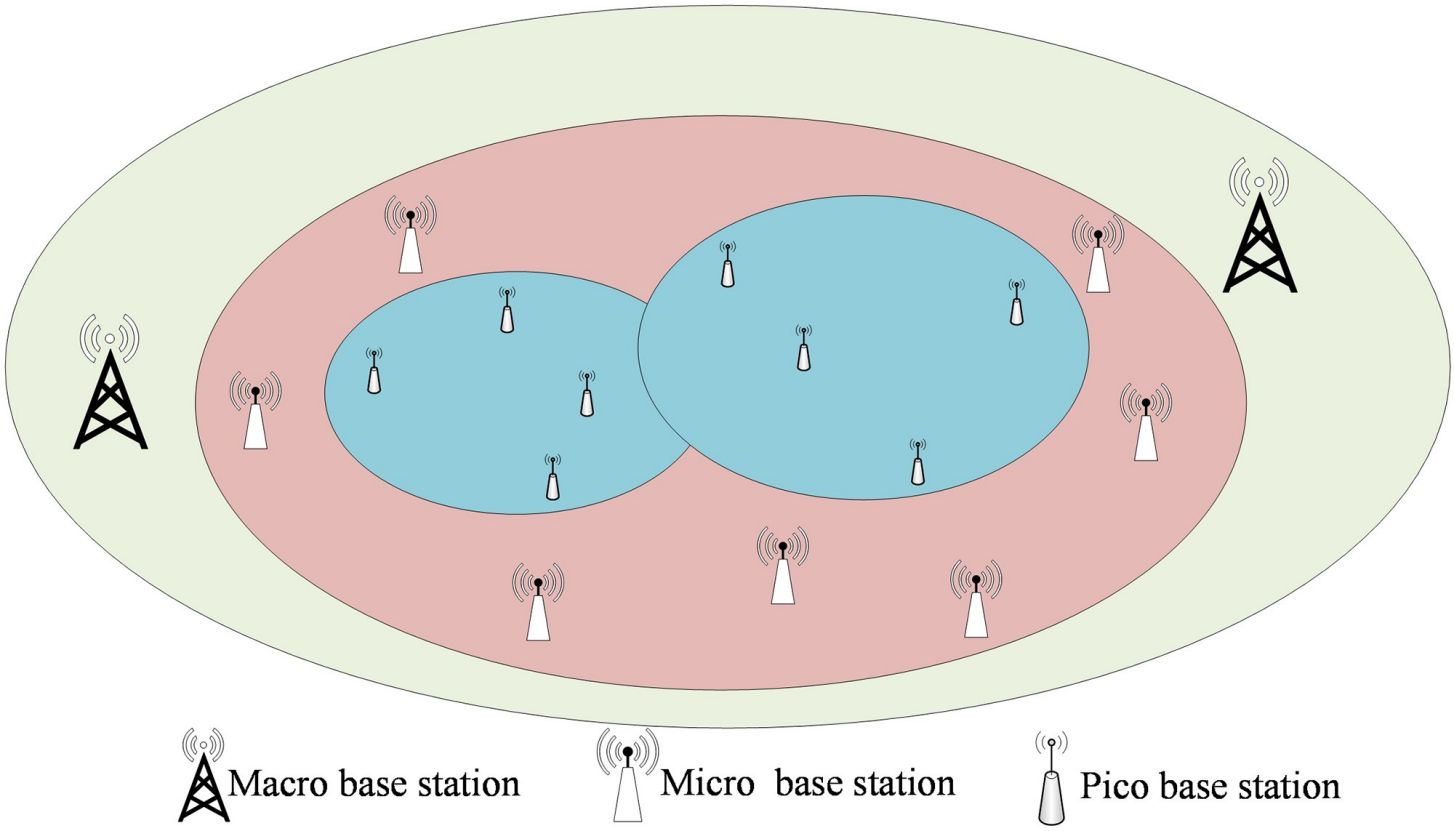
A schematic diagram of the distribution of base stations in a city shows that the distribution of base stations is not even, showing a trend of being dense inside and sparse outside.

Mobile devices need to access the internet through base stations, generating 5G mobile network traffic. However, the distribution of base stations is not uniform. Fig 6 illustrates the deployment scenario of 5G base stations. Macro base stations, characterized by high transmission power and wide coverage, primarily offer fundamental network coverage. On the other hand, low-power micro base stations and pico base stations with limited coverage are strategically positioned in densely populated regions like transportation hubs, commercial areas, and tourist attractions. These smaller base stations help alleviate the load on macro base stations and ensure efficient network performance. This results in diverse and non-uniform distribution of 5G network traffic [35]. Therefore, in order to verify that the model has good generalization ability, data needs to be collected and experiments need to be conducted in various scenarios. We used a laptop, which can access the mobile network by inserting a SIM card, to connect to 5G mobile network and conducted data collection experiments in urban indoor, urban outdoor, and suburban areas from 3:00 to 3:30 pm on three consecutive working days.

During the experiment, we opened two live streams on the laptop and set the video quality to 1080P 60 frames per second. We also started downloading files while limiting the download speed to 8MB/s in order to collect both TCP and UDP traffic data. We collected traffic data using Wireshark in one-second intervals, and used it as experimental data.

To confirm that the collected experimental data was generally error-free, we performed a preliminary inspection by plotting the time on the x-axis and the traffic per second on the y-axis. However, we found that the curve of traffic per second changed too drastically, making it difficult to identify patterns and trends, which is not conducive to accurate prediction modeling. Therefore, we applied an Exponentially Weighted Moving Average (EWMA) to the experimental data. EWMA calculates the local average of a variable based on the sequential increase or decrease of new and old data, and updates the variable based on its historical values, thus eliminating the influence of random fluctuations [36]. Let *v* be variable at time *t*, denoted as *v*_*t*_, and let *θ*_*t*_ be the value of *v* at time *t* without using EWMA. That is, when not using EWMA, *v*_*t*_ = *θ*_*t*_. When using EWMA, the update formula for *v*_*t*_ is as follows:

$$\begin{array}{c} v_{t}=\beta v_{t-1}+(1-\beta )\theta_{t},\;0\leq \beta <1 \end{array}$$ (12)

**Fig 7:**
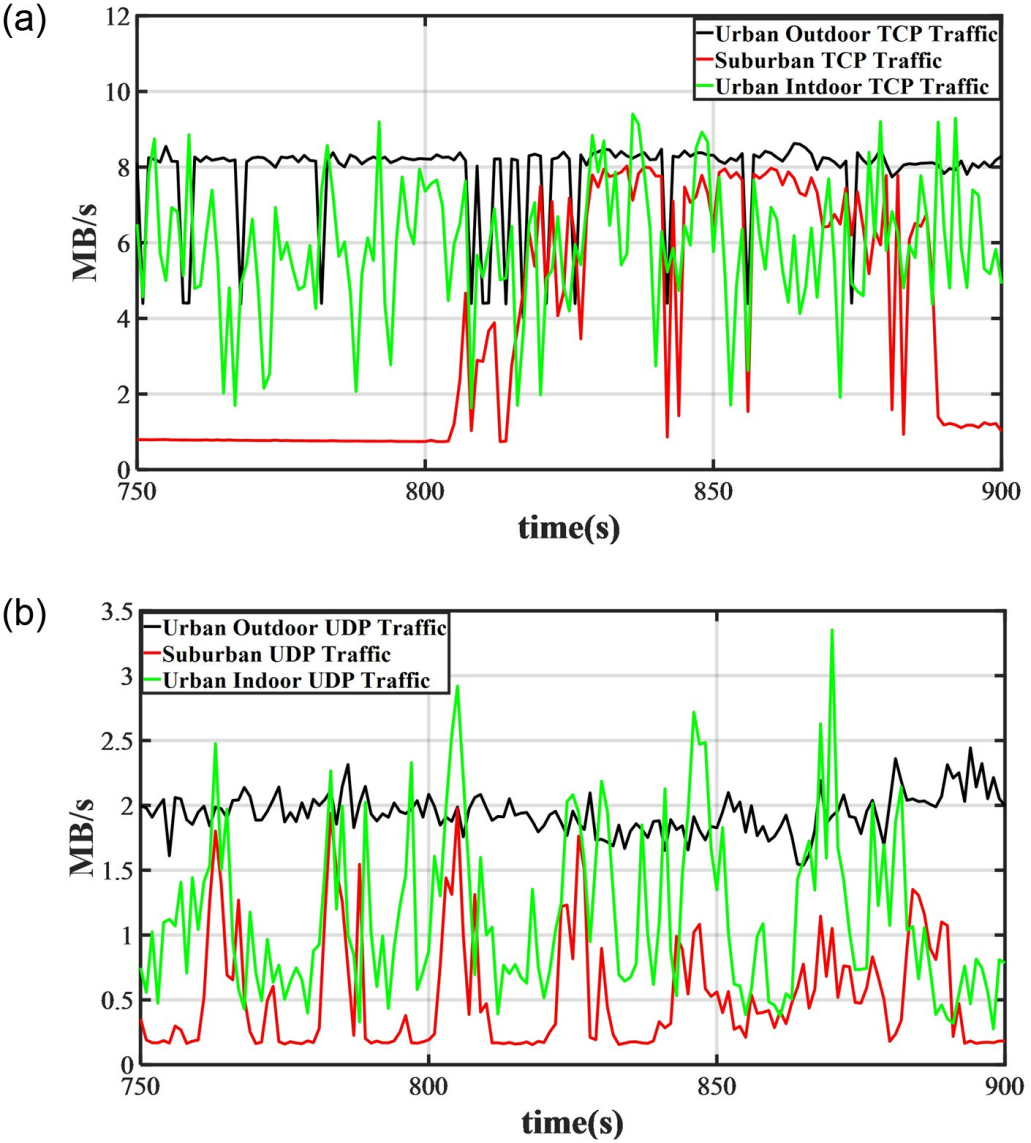
We applied exponential smoothing to the 5G traffic data collected in urban outdoor, urban indoor, and suburban areas, and plotted the TCP and UDP traffic change curves over time. (**a**) TCP traffic sequence, (**b**) UDP traffic sequence.

We set the decay coefficient *β* to 0.2 and used traffic per second as the variable. After applying EWMA, we plotted the time on the x-axis and the traffic per second on the y-axis for the experimental data collected in urban indoor, urban outdoor, and suburban areas. This resulted in Fig 7.

From the graph, it can be seen that in the transmission experiment conducted in urban outdoor areas, TCP traffic remained at around 8MB/s, while UDP traffic fluctuated around 2MB/s. During the experiment, we observed that file downloads could be maintained at the download rate limit, and video playback was generally smooth. This was because the upper limit of the file download rate was set to 8MB/s, and the bandwidth occupied by 1080P 60fps video after video encoding was approximately 1MB/s.

In the transmission experiment conducted in urban indoor areas, TCP and UDP traffic sometimes reached the same level as in the urban outdoor experiment, but most of the time they fluctuated violently. This corresponds to the observation during the experiment that the download speed was usually difficult to maintain at 8MB/s, and video playback sometimes stuttered.

In the transmission experiment conducted in suburban areas, the traffic generated by TCP and UDP traffic was mostly at low values, but TCP traffic occasionally maintained the download rate limit at certain times. The reason for this may be that the location where the experiment was conducted was at the boundary of the base station signal coverage, or because the base station used for information forwarding was not the same due to load balancing.

Comparing the experiments conducted in urban outdoor, urban indoor, and suburban areas, it can be seen that due to the denser distribution of base stations in urban areas, the traffic is larger than that in suburban areas during the same period. Due to the large number of obstacles in the indoor environment in urban areas, severe signal attenuation and interference, traffic fluctuations are larger indoors, while they are smaller outdoors. In suburban areas, due to the low density of base stations and the long distance between devices and base stations resulting in weak signals, traffic is mostly low without the installation of signal amplifiers.

In the three different experimental environments, the 5G traffic showed different trends. Therefore, it is necessary to collect traffic data from various environments to train the model to verify its strong generalization ability.

### 4.2 Validation of system design rationality

The system design encompasses the data augmentation and traffic classifier modules. However, it remains unvalidated whether the time series generator employed for data augmentation generates reasonable sequences. Additionally, the robustness of using the KNN algorithm instead of the DBSCAN clustering algorithm for traffic classification during model predictions has not been thoroughly examined. Next, we will use traffic sequences collected from outdoor urban environments to validate these two outstanding issues.

**Fig 8:**
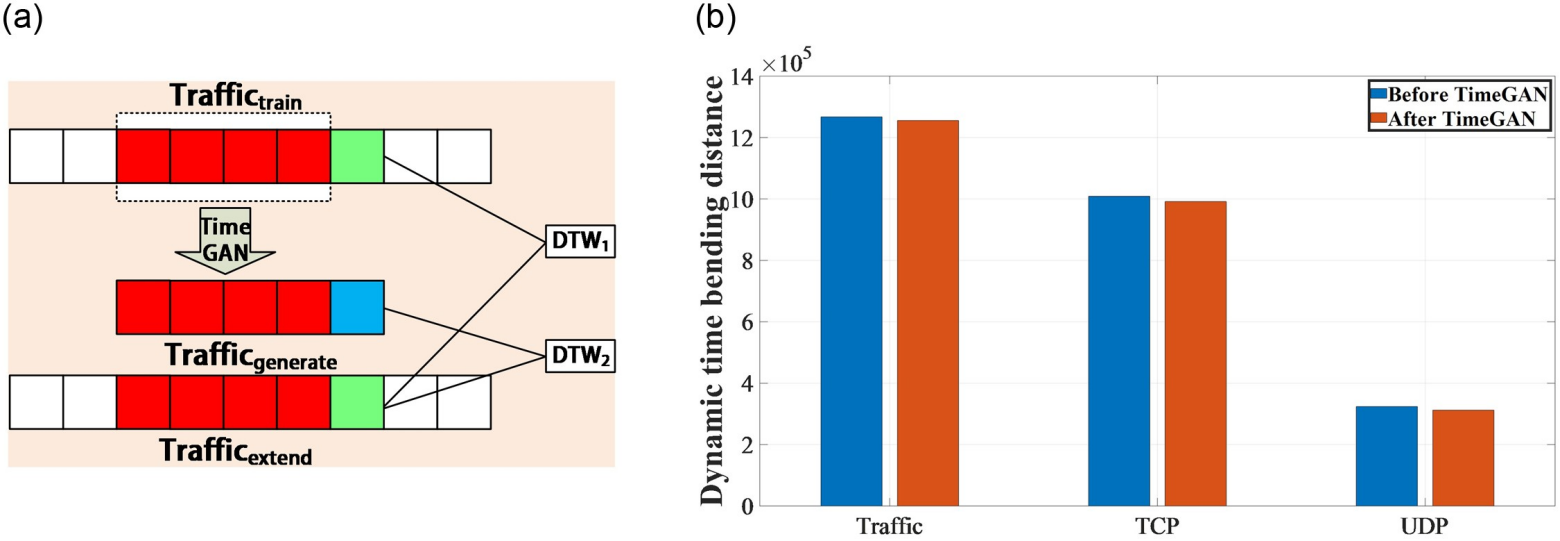
Verification method and verification results of the rationality of time series generator by calculating the similarity of traffic flow sequences. (**a**) Verification method, (**b**) Verification result.

The validation method is shown in Fig 8a. Taking TCP traffic sequence as an example, first a segment of time series is selected from the dataset for training the TimeGAN model, named *Traffic*_*train*_. Then, an additional segment of time series generated by TimeGAN is concatenated with *Traffic*_*train*_ to obtain *Traffic*_*generate*_. Extend the length of *Traffic*_*train*_ in the dataset to the same length as *Traffic*_*generate*_ to obtain *Traffic*_*extend*_. The dynamic time-warping distances of *Traffic*_*extend*_ and *Traffic*_*generate*_, as well as the dynamic time-warping distance of *Traffic*_*extend*_ itself are calculated, denoted as *DTW*_1_ and *DTW*_2_, respectively. The relative change of *DTW*_2_ over *DTW*_1_ is compared to validate the effectiveness of the data augmentation module design.

We extracted the traffic sequence from the 100th to the 500th second obtained from outdoor experiments in urban areas as the training dataset for TimeGAN, which generated the traffic sequence from the 500th to the 600th second used to calculate *DTW*_2_. At the same time, we also extracted the traffic sequence from the 100th to the 600th second obtained from outdoor experiments in urban areas to calculate *DTW*_1_, for comparison. The experimental results we obtained are shown in the following figure.

From the experimental results, it can be seen that the dynamic time-warping distance of the traffic sequence does not change much after being processed by TimeGAN. For example, for the TCP traffic sequence, *DTW*_1_ is about 1.267 × 10^6^, and *DTW*_2_ is 1.255 × 10^6^. The dynamic time-warping distance of TCP traffic only changed by about 1% after being processed by the data augmentation module. This indicates that the time series generated by the TimeGAN model can be almost regarded as an extension of the original time series. At the same time, it also verifies the rationality of using the TimeGAN model as a data augmentation module for data augmentation operations on 5G traffic sequences. Furthermore, it can be observed that the dynamic time-warping distance of the Traffic, TCP, and UDP sequences decrease slightly after being processed by TimeGAN, indicating that the values of the newly generated sequences are generally smaller than those of their corresponding original sequences. In other words, the TimeGAN model is a relatively conservative time series generator, which tends to generate stable time series.

**Fig 9:**
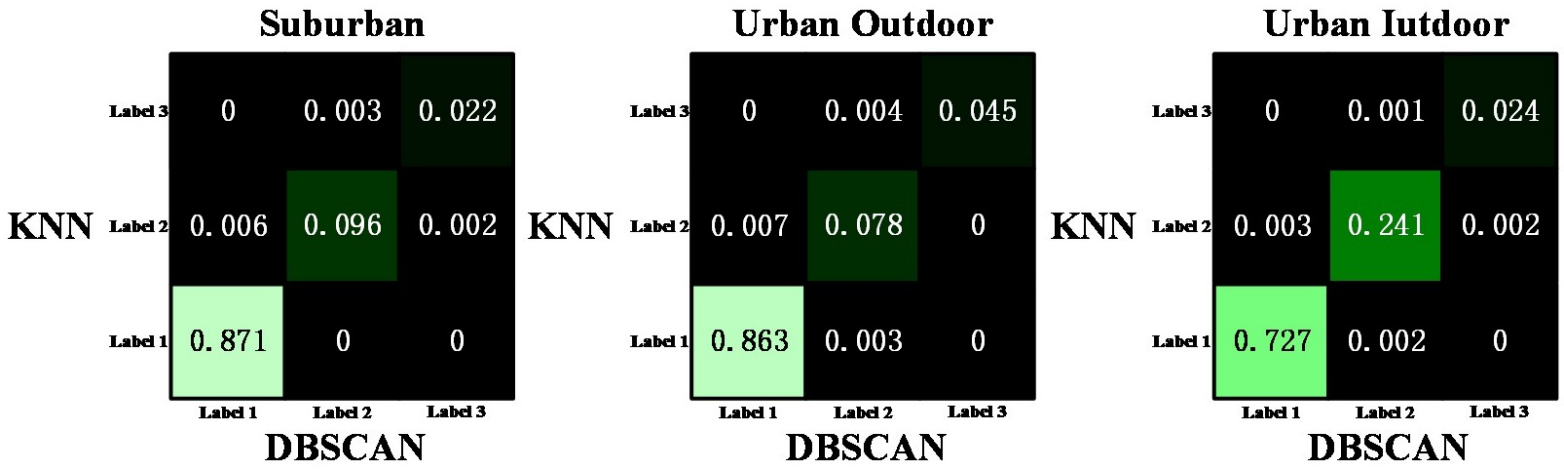
Comparison of traffic data collected in urban indoor, urban outdoor, and suburban through DBSCAN clustering and KNN clustering.

Next, we will verify the robustness of the traffic classifier. Specifically, the traffic classifier uses DBSCAN clustering algorithm during training and KNN clustering algorithm during prediction. Our plan is to initially utilize the DBSCAN algorithm to classify the training set data. Subsequently, based on the classification results obtained from the training set, we will employ the KNN algorithm to classify the test set data. By comparing the classification results of the test set data obtained by DBSCAN, we aim to validate the robustness of the traffic classifier. From Fig 9, it can be seen that the classification results of DBSCAN and KNN based on DBSCAN classification results are almost identical, with only a small portion of traffic data classified differently. This is expected because the test set and training set traffic data are segmented from the same time series, and different parts of the same time series usually exhibit similar trends. In addition, we compare the classification results in three different experimental environments. As shown in Fig 7, traffic is usually lower in suburban environments and higher in urban outdoor environments, with less fluctuation. This is why most traffic data in suburban and urban outdoor environments are classified as Label 1 by both clustering algorithms in Fig 9. Due to the large fluctuation of traffic data in urban indoor environments, a considerable portion of traffic data is classified as Label 2 by both clustering algorithms. However, regardless of the experimental environment, the classification results of both methods are similar, indicating that the traffic classifier designed in this paper is not only robust but also has good generalization.

In this section, we compared the dynamic time-warping distance between the generated sequences and the original sequences, and showed that the additional time series generated by the data augmentation module based on TimeGAN can be considered to come from the same time series as the sampled sequences. In addition, we compared the classification results of two clustering algorithms on test set traffic data in multiple experimental environments. The nearly consistent classification results of the two algorithms also demonstrated the robustness and generalization of the traffic classifier designed in this paper.

### 4.3 Impact of pruning algorithm

**Fig 10:**
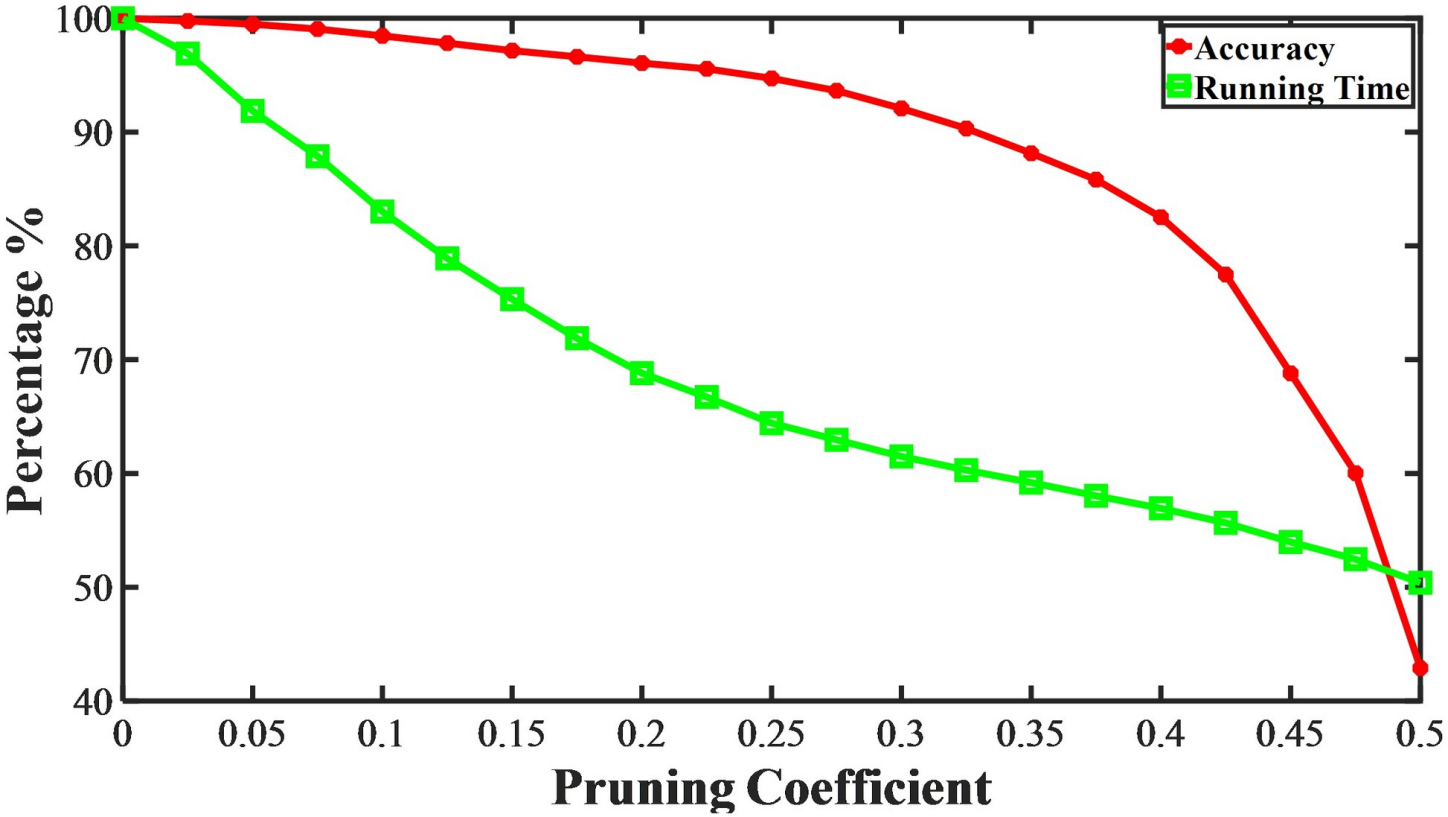
The impact of setting different pruning coefficients on the model accuracy and one-step prediction time of CLPREM after using the pruning algorithm.

In order to reduce the computational complexity of the model and improve its timeliness to meet the needs of real-time prediction, this paper prunes the model. Algorithm 1 shows a heuristic pruning algorithm used in this paper. First, the pre-trained model is used to make the network converge. Then, weights that are lower than the mean and the product of the proportion coefficient are set to 0. Finally, the pruned network is fine-tuned to restore its accuracy. The deep learning network designed in this paper contains multiple layers, and the weight values of different layers need to be divided into different sets for sorting and pruning. After pruning and fine-tuning, some unimportant weights are set to 0, and the data connected to them do not need to participate in the calculation. This reduces the computational complexity of the model and improves its timeliness. From Fig 10, it can be seen that the pruning algorithm does compress the model, reduce running time, and improve its timeliness. However, it also brings the problem of model accuracy reduction. Therefore, it is necessary to choose a suitable pruning coefficient to ensure that the model is compressed without losing too much accuracy. It can be seen that when 0 < *θ* < 0.25, increasing the pruning coefficient can significantly reduce the running time of the model, while the reduction speed slows down when 0.25 < *θ* < 0.5. The decrease in model accuracy can be divided into three stages: the accuracy reduction is slow when 0 < *θ* < 0.25, it becomes faster when 0.25 < *θ* < 0.35, and it drops rapidly when 0.35 < *θ* < 0.5. It can be seen that when *θ* = 0.25, the accuracy only decreases by about 5%, but the running time of the model has already been reduced to 65% of the original. Therefore, we believe that the optimal pruning coefficient for CLPREM is 0.25, and the subsequent prediction model is also based on the pruning operation with a pruning coefficient of 0.25 and fine-tuned accordingly.

### 4.4 Performance of CLPREM in different environments

We trained CLPREM using 70% of the collected data in three different environments, and the results presented in this section were obtained by testing the model on the remaining 30% of the training set. According to the pruning experiments in Section 4.3, we set the sparsity coefficient to 0.25. Additionally, to prevent overfitting, we added a regularization term to the model and set the regularization coefficient to 0.1. After completing the training, we input 5 seconds of traffic data to the model at once, with a batch size of 5. The model predicts the next 5 seconds of traffic changes based on the input batch of data, and the prediction step size is also 5. More detailed Settings for CLPREM are shown in Table 1.

**Table 1:** The hyperparameters selected for CLPREM, as well as other settings of the model, were determined based on experience and testing.

Hyperparameters Setting		Other Setting	
Epochs	64	Activation Functions	Sigmoid
Learning Rate	10e-3	Optimization Algorithm	Adam
Batch Size	10	Weight Initialization	Random Uniform
Pruning Coefficient	0.25	Normalization Method	Z-score
Regularization	0.1		
The number of hidden layer neurons	12		
Training Percentile	0.7		

**Fig 11:**
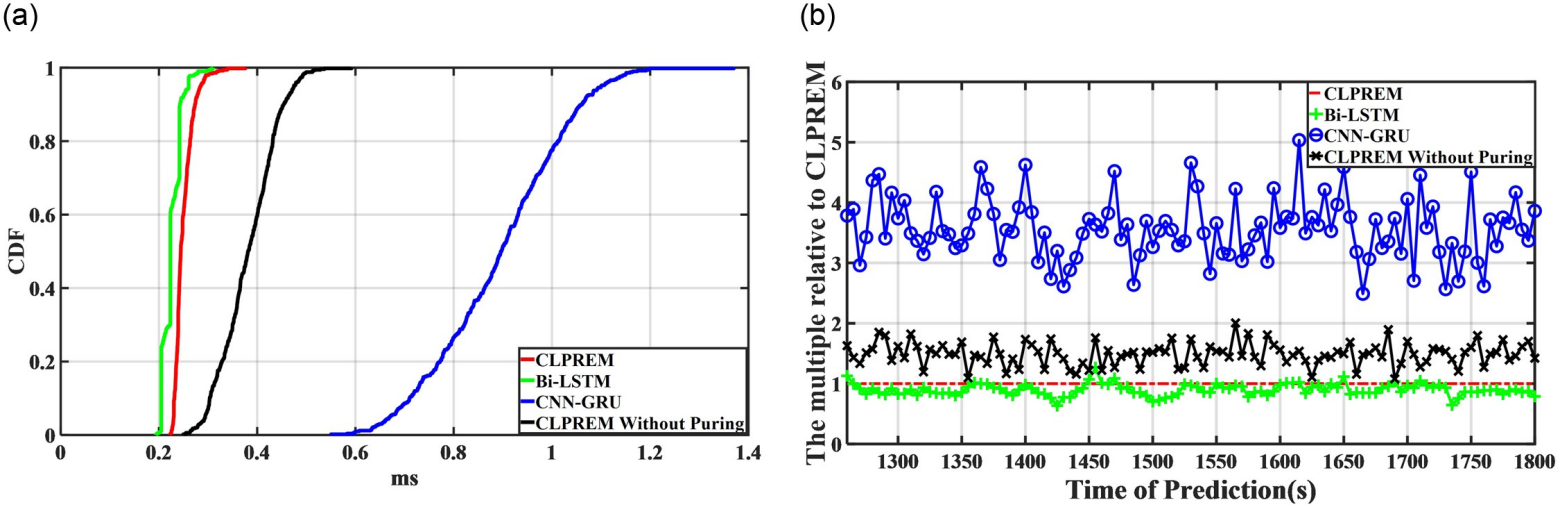
We recorded the distribution function of the one-step prediction time for CLPREM and its comparative models, as well as a curve graph showing the multiple of the comparative model’s prediction time relative to CLPREM. Each sample in the distribution function is obtained by averaging the results in the three environments. (**a**) Prediction time distribution function, (**b**) Multiple of the running time.

We also trained models using Bidirectional Long Short-Term Memory (Bi-LSTM), combination of Convolutional Neural Networks and Gated Recurrent Units (CNN-GRU), and CLPREM before pruning, following the same methodology, to compare their predictive performance with CLPREM. Initially, we planned to keep the parameters of these models consistent with CLPREM. However, during actual testing, we found that even after increasing the epoch to 128, the Bi-LSTM model was still underfitting when we set the number of hidden states in the LSTM layer to 12. We had to increase the number of hidden states in Bi-LSTM to 24 to avoid significant errors in its predictions. Under these circumstances, we first tested the one-step prediction, in which the model predicts the next 5s of network traffic data based on 5s of input network traffic data after completing the training. We averaged the predicted time for each corresponding time in the three environments and obtained the test results shown in Fig 11.

From Fig 11a, it can be seen that the one-step prediction time of CLPREM is approximately between 0.2–0.4ms, and the vast majority of the prediction time is within 0.3ms, which means that CLPREM can complete the prediction of future 5s traffic with only 0.3ms of time, indicating that CLPREM has good efficiency. However, by observing the distribution function of CLPREM and Bi-LSTM, it can be seen that although the hidden state of Bi-LSTM is two times that of CLPREM, Bi-LSTM is still slightly faster than CLPREM. This is because CLPREM actually consists of three LSTMs, and there are still weight connections between the LSTMs, in addition to the KNN classification and two hidden layers, which makes the prediction efficiency of CLPREM slightly lower than that of Bi-LSTM. By comparing the distribution function curve of CLPREM before and after pruning, it can be seen that pruning has indeed shortened the model’s one-step prediction time and improved its efficiency. Compared with CNN-GRU, we can see that whether or not CLPREM is pruned, it is significantly faster than CNN-GRU. This is because although the parameter settings of GRU are similar to CLPREM, the theoretical prediction time should be slightly shorter than CLPREM using LSTM. However, in CNN-GRU, the prediction of GRU depends on the feature extraction of the CNN network, and the feature extraction process consumes a lot of time, ultimately leading to the lower efficiency of the CNN-GRU model compared to CLPREM [37].

Fig 11b shows the time for each model to make each prediction, providing clearer and more visible data on prediction time. The prediction time lasts from 1260s to 1800s. Overall, the prediction time of Bi-LSTM is almost similar to that of CLPREM, and pruning also reduces CLPREM’s prediction time by nearly one-third. Compared to the longest prediction time of CNN-GRU, the prediction time of CLPREM is almost only one-fourth of it.

We have compared the differences in prediction time between CLPREM and its comparative models, but for the real-time prediction problem of 5G traffic, a model cannot be considered good just because it has a short prediction time. Further comparison of real-time prediction accuracy and generalization ability is needed.

**Fig 12:**
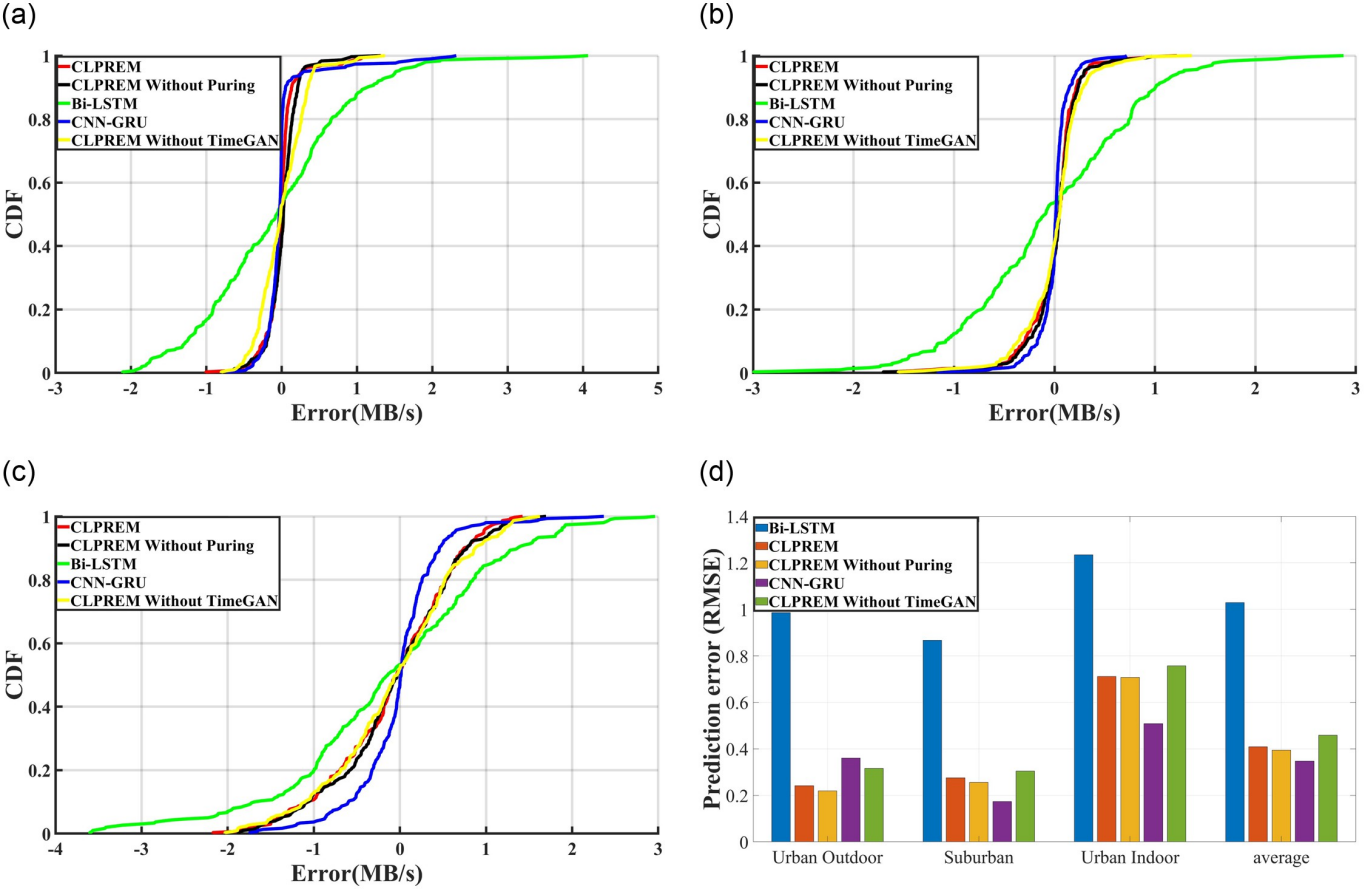
The distribution function graphs of prediction errors for CLPREM model and its comparative models in three different environments, as well as the root mean square error of predictions by CLPREM model and its comparative models in each environment. (**a**) Urban outdoor (**b**) Suburban, (**c**) Urban indoor, (**d**) Prediction error of four methods.

To demonstrate that CLPREM has good generalization ability, we cannot simply average the prediction time in the three scenarios, as we did with the comparison of prediction time. This is because a model can only prove to have good generalization ability if it has accurate predictions in multiple scenarios. Therefore, we have plotted the distribution function of the prediction accuracy of CLPREM and its comparative models in three different scenarios, as shown in Fig 12.

Fig 12 shows the prediction accuracy of CLPREM and its comparative models in outdoor urban, suburban, and indoor urban environments. Firstly, it is evident that even though the number of neurons in Bi-LSTM is twice that of other models, its prediction accuracy is still poor. In fact, we trained Bi-LSTM several times, but in most cases, it did not learn the features of 5G network traffic, resulting in a prediction curve that was almost a straight line. The plot presented here is a relatively good set of data selected from multiple prediction experiments. This implies that even though the LSTM layer of Bi-LSTM has more hidden states than other models, its ability to learn 5G traffic features is still not strong enough. Although it has a shorter prediction time, its poor performance makes it unsuitable for real-time prediction of 5G traffic.

Fig 12 also includes a comparison of whether TimeGAN was used for data augmentation on the time series, and it can be observed that data augmentation has improved the prediction accuracy to some extent. This may be due to TimeGAN generating time series with similar characteristics, expanding the dataset, and enabling CLPREM to capture more variations in the traffic sequence during training at the same epoch.

For the remaining CLPREM and the other two comparative models, we can see that all three models can relatively accurately predict changes in traffic in real-time, with prediction errors within 1MB/s in any scenario, indicating that all three models are highly accurate and have strong generalization ability. However, CLPREM has the shortest prediction time and the best timeliness among the three models. Therefore, compared to other models, CLPREM is more suitable for solving the real-time prediction problem of 5G traffic in different scenarios.

In Fig 12, apart from comparing the prediction accuracy of four models, we also observed that as the complexity of the scene increases and traffic fluctuations become more intense, the prediction accuracy of CLPREM slightly decreases. This suggests that CLPREM is not flawless and there is still potential for further improvement. Undoubtedly, collecting more relevant traffic sequence information as input to the model can further reduce prediction errors. Because the upper limit of the prediction accuracy of deep learning models is often related to the input features of the model, extracting more features usually has a higher upper limit, and adjusting parameters can promote the model to approach this limit. However, extracting more features means that the model needs to spend more time processing, and the timeliness will inevitably decrease. Next, we will try a new time series normalization method to further improve the accuracy of CLPREM without increasing the input data of the model.

In the 4th Makridakis Competition, S. Smyl et al. integrated multiple dilated LSTM models combined with exponential smoothing to forecast time series data from various domains and industries, such as sales data, economic indicators, and weather data [38]. In addition to presenting valuable predictive models, Smyl et al. also raises the question of whether it is appropriate to simply normalize the sequence to [0, 1] as most studies have done. This is because the values to be predicted may fall outside this range, and more importantly, for highly volatile sequences, the smaller values may be ignored after normalization, resulting in a loss of prediction accuracy [38].

The preprocessing method not only overcomes the above problems but also successfully improves the accuracy of their model predictions, and won the first place in the 4th Makridakis Competition. This method is very simple and will not add too much computational pressure to the model, making it suitable for improving the accuracy of CLPREM. It can be simply described as: during training and prediction, each value in the input window is normalized by dividing by the last value in the window, and then a logarithmic function is applied as a compression function to prevent abnormal values from causing too much interference to the model’s learning features. The expression for restoring the predicted data is as follows:

$$\begin{array}{c} {\hat{y}}_{t+1..t+h}=\;exp\left(NN\left(x\right)\right)\;\times \;l_{t} \end{array}$$ (13)

In our experiment, *x* represents the preprocessed traffic data, *NN* represents CLPREM, *l*_*t*_ is the last value in the current time input data, and *h* is the prediction step. Since the preprocessing method obviously did not add too much computational burden, this paper no longer compares the time consumption with or without preprocessing. Next, the main work is to apply this preprocessing method to CLPREM and compare the accuracy improvement brought by preprocessing after each training.

**Fig 13:**
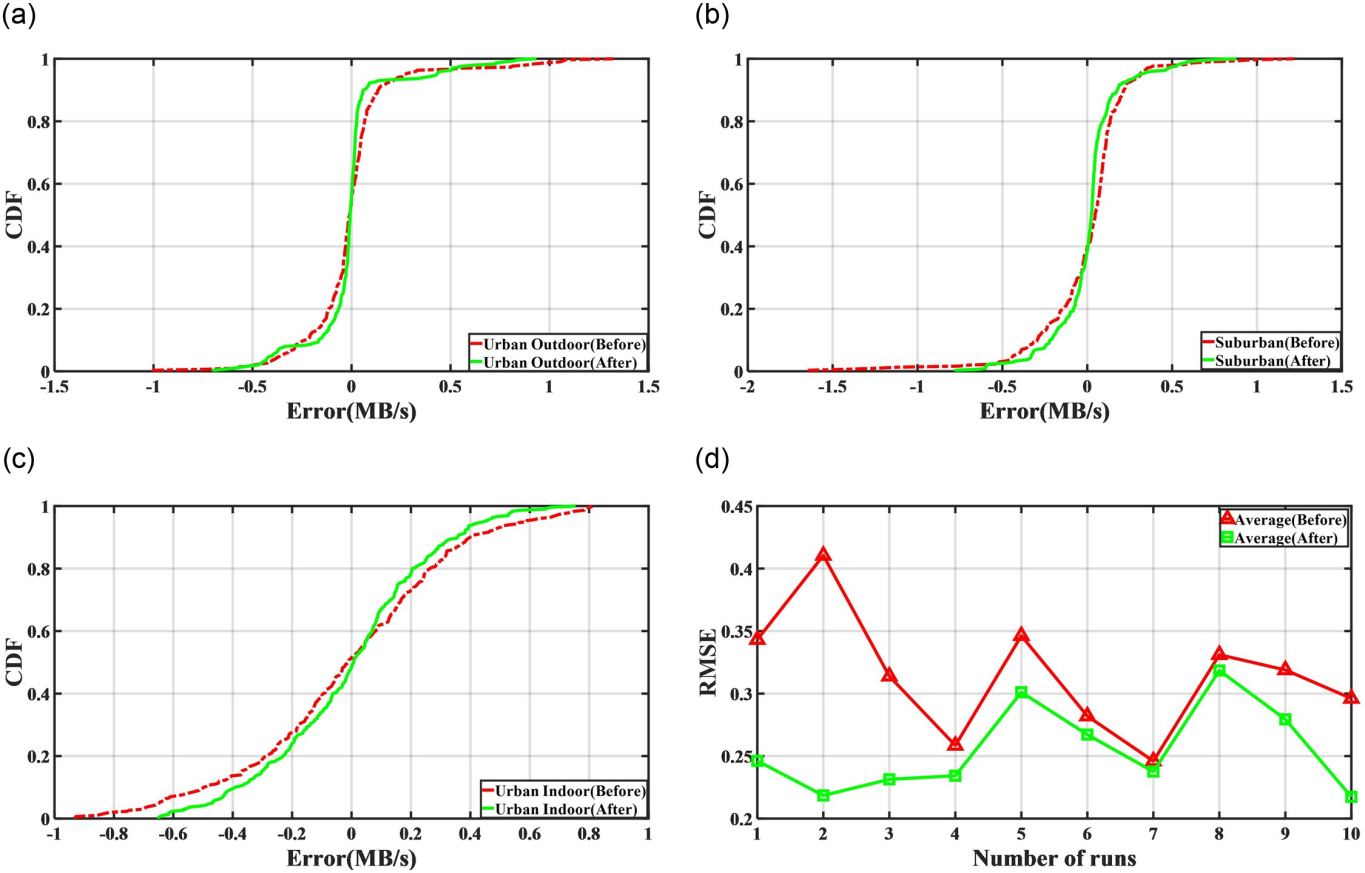
The comparison of results before and after using the preprocessing method is presented, including the distribution functions of the average prediction error in 10 runs under three environments, and the average root mean square error under three environments. (**a**) Urban outdoor, (**b**) Suburban, (**c**) Urban indoor, (**d**) Prediction error.

Since deep learning algorithms initialize neural networks with random numbers at the beginning of training, one of the purposes is to obtain a result with stronger generalization ability [39]. Therefore, with the same parameter settings, different results may be obtained when training CLPREM due to the different random initialization of the network. If only the results before and after using preprocessing are compared for a single time, it is difficult to eliminate randomness, so we conducted ten consecutive prediction experiments, took the average prediction error, and recorded the root mean square error of each prediction. The results shown in Fig 13 were obtained.

In the comparative experiment in Fig 12, we have already found that CLPREM’s distribution function has a tail phenomenon, especially in the suburban environment prediction experiment. This indicates that CLPREM’s prediction will have a large prediction error in a few times, which is also the main source of the root mean square error. From Fig 13, even if ten consecutive predictions are taken and averaged, this tail phenomenon of CLPREM still remains. However, after applying the preprocessing method, it can be seen that the tail phenomenon of CLPREM’s distribution function has been alleviated. This is also the main reason why the root mean square error after using preprocessing is always smaller than that without preprocessing in ten consecutive predictions.

The reason why this preprocessing method improves the performance of CLPREM can be explained. If the previous global normalization method is adopted, for a time period with small fluctuations in the time series, the normalized sequence will not show significant changes, and the model will also have difficulty learning its features [40]. For time periods with large fluctuations, the predicted values may be outside [0, 1], which will also lead to errors. Moreover, the preprocessing method designed also reduces the impact of outliers on the model by taking logarithms.

As shown in Fig 7, the changes in 5G traffic in different scenarios show different trends, with relatively stable performance in urban outdoor areas, frequent fluctuations in urban indoor areas, and mixed results in suburban areas. It is because of the diverse changes in 5G traffic that we decided to adopt the preprocessing method. The results are consistent with our expectations, and this method does improve the predictive accuracy of the model without increasing the time overhead.

## 5 Conclusion

This paper proposes a 5G traffic real-time prediction scheme CLPREM, which can be applied to network resource optimization, anomaly detection, and other aspects. Based on the LSTM network, CLPREM distinguishes network behaviors by applying clustering algorithms, enhances the model’s robustness by applying data augmentation algorithms, and achieves model light weighting by applying pruning algorithms. Unlike traditional prediction methods, CLPREM has strong generalization ability and can work in various environments. By analyzing current network behavior and historical traffic information, CLPREM completes real-time traffic prediction. To verify the rationality of the data augmentation and clustering modules of CLPREM, we designed two clever experiments, and the results also proved the rationality of the design. To determine the pruning coefficient, we tested the accuracy and prediction time of the model under different pruning coefficients, and analyzed the trend of accuracy and prediction time with the increase of pruning coefficients to determine the pruning coefficient used in subsequent experiments. To test the overall performance of CLPREM, we compared the prediction accuracy and prediction time of CLPREM with other algorithms based on traffic data collected in three environments: urban outdoor, urban indoor, and suburban areas. The results show that CLPREM can achieve the accuracy of the CNN-GRU model with time overhead close to the Bi-LSTM model, and its stable performance in the three environments also proves its strong generalization ability. Finally, we adopted a preprocessing method that almost does not increase the time overhead, further improving the accuracy of CLPREM and effectively solving the tail problem of CLPREM prediction. The source code for CLPREM is available at https://github.com/FishT0ucher/CLPREM/tree/main/CLPREM.

Although CLPREM has achieved promising results in real-time traffic prediction for 5G networks, further in-depth research in this field is still warranted, with a primary focus on the following two aspects:

1. Optimization of model architecture: One future direction is to optimize the architecture of CLPREM. Advanced deep learning techniques, such as attention mechanisms, can be explored to capture more complex dependencies in network traffic data. Additionally, more powerful model compression techniques can be considered to improve the model’s timeliness.
2. Incorporation of additional relevant factors: Traffic variations are influenced by various external factors. Future research can explore the incorporation of more external factors into the prediction model, such as network topology and user behavior.

In summary, future work should be dedicated to further enhancing the timeliness, accuracy, and robustness of CLPREM, as well as applying it to a wider range of practical scenarios to meet the demands of real-time traffic prediction for 5G networks.

## Supporting information

S1 File (DOCX)
