# Supplementary material for: CLPREM: A real-time traffic prediction method for 5G mobile network
Source: PLoS One. 2024 Apr 1;19(4):e0288296. doi: 10.1371/journal.pone.0288296 (PMC10984461; doi:10.1371/journal.pone.0288296)
Supplement: S1 File — (DOCX) [file pone.0288296.s001.docx]

Figure：

https://github.com/FishT0ucher/Figure/tree/main/plos%20one%20figure

Code & Data：

https://github.com/FishT0ucher/CLPREM/tree/main/CLPREM
